# Selection of three miRNA signatures with prognostic value in non-M3 acute myeloid leukemia

**DOI:** 10.1186/s12885-019-5315-z

**Published:** 2019-01-30

**Authors:** Yao Xue, Yuqiu Ge, Meiyun Kang, Cong Wu, Yaping Wang, Liucheng Rong, Yongjun Fang

**Affiliations:** 1 Department of Hematology and Oncology, Children’s Hospital of Nanjing Medical University, Nanjing, China; 2 Key Laboratory of Hematology, Nanjing Medical University, Nanjing, China; 3 Department of Public Health and Preventive Medicine, Wuxi School of Medicine, Jiangnan University, Wuxi, China; 4 Engineering Research Center of Wideband Wireless Communication Technology, Ministry of Education, Nanjing University of Posts and Telecommunications, Nanjing, China

**Keywords:** Acute myeloid leukemia, Prognosis, miRNAs, TCGA data

## Abstract

**Background:**

MiRNAs that are potential biomarkers for predicting prognosis for acute myeloid leukemia (AML) have been identified. However, comprehensive analyses investigating the association between miRNA expression profiles and AML survival remain relatively deficient.

**Method:**

In the present study, we performed multivariate Cox’s analysis and principal component analysis (PCA) using data from The Cancer Genome Atlas (TCGA) to identify potential molecular signatures for predicting non-M3 AML prognosis.

**Result:**

We found that patients who were still living were significantly younger at diagnosis than those who had died (*P* = 0.001). In addition, there was a marked difference in living status among different risk category groups (*P* = 0.022). A multivariate Cox model suggested that three miRNAs were potential biomarkers of non-M3 AML prognosis, including miR-181a-2, miR-25 and miR-362. Subsequently, PCA analyses were conducted to comprehensively represent the expression levels of these three miRNAs in each patient with a PCA value. According to the log-rank test, AML outcome for patients with lower PCA values was significantly different from those with higher PCA values (*P* < 0.001). Further bioinformatic analysis revealed the biological functions of the selected miRNAs.

**Conclusion:**

We conducted a comprehensive analysis of TCGA non-M3 AML data, identifying three miRNAs that are significantly correlated with AML survival. PCA values for the identified miRNAs are valuable for predicting AML prognosis.

**Electronic supplementary material:**

The online version of this article (10.1186/s12885-019-5315-z) contains supplementary material, which is available to authorized users.

## Background

Acute myeloid leukemia (AML) is the most common acute leukemia in adult patients and is an aggressive haematological malignancy. It is a haematological malignancy that is promoted by various factors, including environmental exposure and several abnormality in cellular and molecular level [1]. Despite decades of effort and achievements, the detailed mechanism of AML remains unclear. Studies have demonstrated that several biological factors may be involved in the pathogenesis and clinical outcomes of AML. As determining patient prognosis is important for individualized treatment, it is essential to comprehensively investigate biological factors that may be associated with AML outcomes and to select valuable biomarkers for prognosis prediction.

MicroRNAs (miRNAs), small noncoding RNA species approximately 22 nucleotides in length, have been demonstrated to play a predominant role in translational and post-transcriptional regulation. miRNAs participate in a wide range of biological processes, and their expression levels are frequently abnormal in human cancers [2]. MiRNAs are involved in the pathogenesis of AML and influence the differentiation, proliferation of haematopoietic cells and clinical treatment response [3–5]. Expression levels of miRNA have been identified as potential biomarkers for predicting prognosis in various human cancers, including AML [6–9]. However, most studies have focused on one or few miRNAs, while synthesizing several miRNA signatures to comprehensively predict AML outcome has yet to be done.

In most cases of AML, patients at the same clinical stage may experience completely different clinical outcomes. Therefore, there is a critical need to explore novel biomarkers that facilitate the clinical prediction of AML prognosis. In 2013, The Cancer Genome Atlas (TCGA) Research Network reported an analysis of the genomes of 200 AML cases, along with RNA and microRNA sequencing data. They found that miRNA signatures were associated with unfavourable, intermediate, and favourable cytogenetic risk categories [10]. Herein, we performed comprehensive analysis using data from TCGA, which contains200 AML patients and expression data for705 miRNAs. We sought to identify potential molecular signatures to predict non-M3 AML prognosis. In addition, bioinformatics analyses were performed to evaluate functions of the identified miRNAs. Our results aid in further characterizing mechanisms of non-M3 AML pathogenesis.

## Methods

### AML dataset in the TCGA database

MiRNA-seq data for 705 miRNAs and clinical data for AML patients were downloaded from the TCGA data portal (https://portal.gdc.cancer.gov/), an interactive data system for researchers to query, download, upload, and analyse harmonized cancer genomic data sets. Since M3 subtype has different biologic features and favourable outcome from other AML subtypes, we only included 179 non-M3 patients in the present analysis. Patients were treated in accordance with National Comprehensive Cancer Network (NCCN) guidelines (www.nccn.org). Many unfavourable risk and intermediate risk patients underwent allogeneic transplantation at some point in their disease course. RNA was extracted from whole blood, and patient samples were collected prior to treatment. Patients without miRNA expression data or clinical information were excluded. As a result, there were 147 non-M3 AML patients included in our study. Clinical demographics (e.g., gender, age, risk category) and survival data (overall and event-free survival) for these patients were also obtained from the TCGA dataset.

### Multivariate survival analysis

We used clinical information, including age, gender, white blood cell (WBC) count to build a multivariate Cox proportional hazard ratio model. Clinical parameters were selected as significant using 0.05 as the cut off. miRNA with missing expression data > 75% were deleted for the following analyses. A multivariate Cox model was used to evaluate the association between expression level of each miRNA and prognosis of a patient. Subsequently, false discovery rate (FDR) adjustment was conducted, and significant miRNAs were filtered out with a cut off of 0.05. As a result, we screened out three miRNAs that were significantly related to survival time in AML as potential biomarkers of prognosis. Subsequently, One-way ANOVA test was performed to investigate whether there was significant difference of miRNA expression level among different sub-groups of AML.

### Analyses of PCA and the association between miRNA expression and AML survival

To determine whether selected miRNAs were reliable indices for clinical prediction of AML prognosis, we conducted principal component analysis (PCA) to obtain a value for each patient based on the expression level of these miRNAs. In addition, the median of all the PCA values was used as a cut off to separate patients into one of two groups. We performed the log-rank test to investigate whether there was a significant difference between the survival of the two groups, and we used Kaplan-Meier plots to visualize survival curves.

### Hierarchical clustering analysis

Patients were divided into two groups according to their survival status (i.e., non-censored patients were recognized as the poor-outcome group, while censored patients denoted the good-outcome group). We performed unsupervised hierarchical clustering analysis using the Cluster/Tree View program to give a general overview of the expression of the selected miRNAs in the good-outcome and poor-outcome groups. A heat map was created with expression levels of the three miRNAs and grouping information from AML patients to visualize the relationship between miRNA expression level and overall survival (OS).

### Integrated gene expression

We also downloaded RNA-sequencing data for AML patients from the TCGA database. A total of 135 cases with both coding gene expression levels and miRNA expression data were included in subsequent correlation analyses. We performed a Pearson correlation for each miRNA and all the coding genes and then selected the top 100 coding genes with the highest Pearson correlation coefficient. The top 100 genes were considered potential targets of the miRNAs because their expression levels were most correlated with those of the miRNAs.

### Gene ontology (GO) analysis

We used the online software DAVID (Database for Annotation, Visualization and Integrated Discovery, http://david.abcc.ncifcrf.gov/home.jsp) to analyse the molecular functions and biological pathways of the coding genes most correlated with the selected miRNAs (top100 of Pearson correlation coefficient). Names of the correlated coding genes were input into DAVID, and the results showed an annotation of input genes utilizing Gene Ontology (GO) terms (http://www.geneontology.org/).

## Results

### Association between clinical factors and AML survival

Relative data were downloaded from TCGA database. Patients without miRNA expression data or clinical information were excluded. As a result, 147 non-M3 AML patients were included in our study. Among them, 96 were non-censored while the other 51 patients were censored. We subsequently investigated the association between patient clinical characteristics and survival status. As shown in Table 1, patients who were still living were significantly younger at diagnosis than those who were deceased (49.2 ± 14.4 VS. 58.2 ± 16.2, *P* = 0.001). In addition, there was a remarkable difference in living status among different groups of risk category (*P* = 0.022). However, we did not find any differences in the proportion of living patients between males and females (*P* = 0.932). Furthermore, the distribution of survivors among different FAB classifications of AML were not significantly different (*P* = 0.767).

**Table 1:** Clinical characteristics of the included 147 non-M3 AML patients and the their survival status

Characteristics	Deceased (*n* = 96)	Living (*n* = 51)	*P* value
Age	58.2 ± 16.2	49.2 ± 14.4	**0.001**
Gender			
Male	52	28	0.932^*^
Female	44	23	
Risk category ^a^			
Favorable	7	12	**0.022** ^*^
Intermediate	64	30	
Poor	23	9	
FAB morphology^b^			
M0 Undifferentiated	8	5	0.767^*^
M1	23	13	
M2	22	14	
M4	27	12	
M5	11	6	
M6	2	0	
M7	3	0	

Boldface represent *P* < 0.05

^*^P value of chi-square test

^a^ Risk information of two cases were missing in the TCGA data

^b^FAB morphology information of one living patient was missing

### Selection of miRNAs significantly associated with AML prognosis

**Fig. 1:**
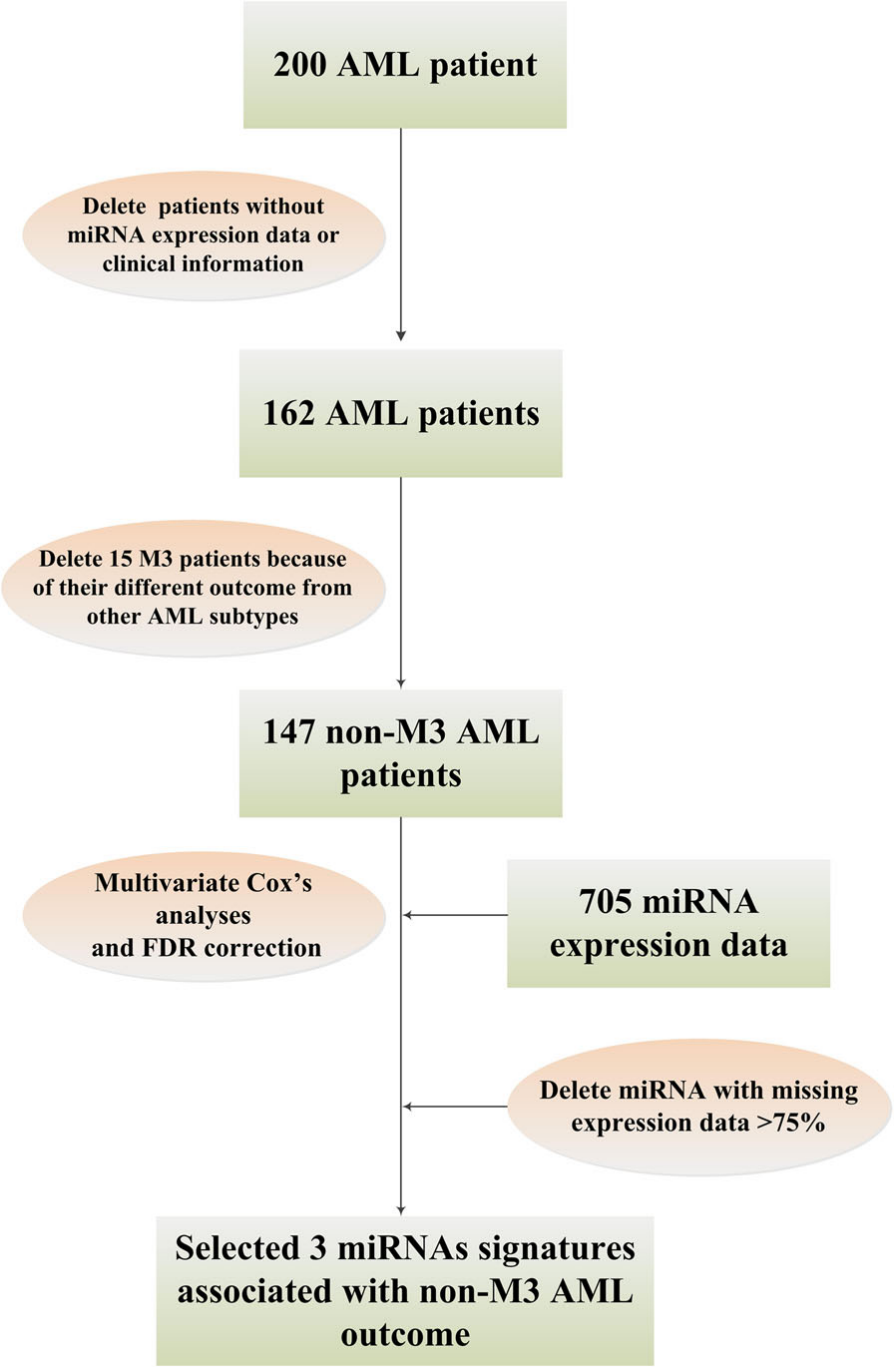
The workflow of carrying out the 3 miRNA signature

After conducting multivariate Cox analyses of expression levels of all the miRNAs and clinical OS of AML cases, we selected three miRNAs as biomarkers of AML prognosis because their expression levels were significantly associated with OS time and survival status, with an FDR correlation less than 0.05. As shown in Table 2, higher levels of hsa.mir.362 indicated poorer prognosis (i.e. hazard ratio > 1), while higher levels of hsa.mir.25 and hsa.mir.181a.2 were related with better outcome (i.e. hazard ratio < 1). The workflow of carrying out the 3 miRNA signature was provided in Fig. 1 and a list of the top 50 miRNAs in the multivariate analysis after adjustment by FDR is shown in Additional file 1: Table S1.

**Table 2:** Three miRNAs were identified to be associated with AML survival

miRNAs	*P* value	FDR	Hazard ratio
miR-181a-2	2.52E-04	4.69E-02	0.45
miR-25	3.98E-04	4.69E-02	0.46
miR-362	3.84E-04	4.69E-02	2.13

**Fig. 2:**
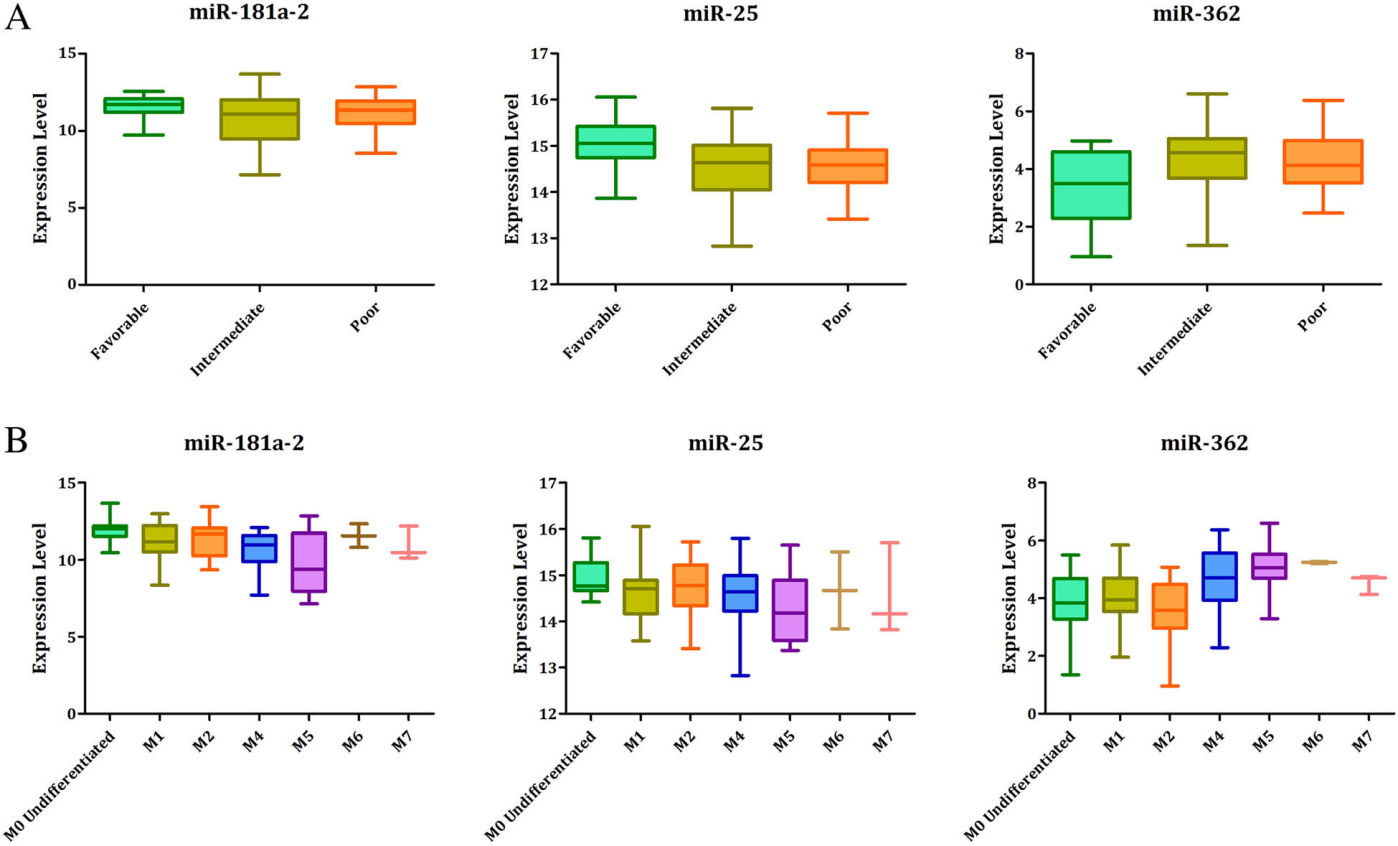
Expression level of three selected miRNA in different AML subgroup (**a** AML risk groups; **b** FAB subtype of AML)

In addition, we also found that expression of these miRNA was remarkable different among different AML subgroup (shown in Table 3 and Fig. 2), indicating a potential biological role of these selected miRNAs in development of AML.

**Table 3:** Expression of three selected miRNA among different AML subgroup

	miR-181a-2	*P* ^*^	miR-25	*P* ^*^	miR-362	*P* ^*^
Risk category ^a^						
Favorable	11.58 ± 0.71	0.046	15.08 ± 0.55	0.004	3.32 ± 1.22	0.001
Intermediate	10.78 ± 1.54		14.55 ± 0.66		4.36 ± 1.09	
Poor	11.15 ± 0.98		14.59 ± 0.54		4.29 ± 0.96	
FAB morphology^b^						
M0 Undifferentiated	11.94 ± 0.73	< 0.001	14.95 ± 0.41	0.079	3.88 ± 1.11	< 0.001
M1	11.12 ± 1.32		14.61 ± 0.61		3.88 ± 1.15	
M2	11.34 ± 1.13		14.77 ± 0.60		3.52 ± 1.07	
M4	10.66 ± 1.17		14.54 ± 0.65		4.73 ± 1.02	
M5	9.70 ± 1.99		14.27 ± 0.70		5.02 ± 0.77	
M6	11.57 ± 1.07		14.67 ± 1.18		5.25 ± 0.04	
M7	10.93 ± 1.09		14.57 ± 1.00		4.53 ± 0.35	

^a^Risk information of two cases were missing in the TCGA data

^b^FAB morphology information of one living patient was missing

### MiRNA biomarkers and their association with AML survival status

**Fig. 3:**
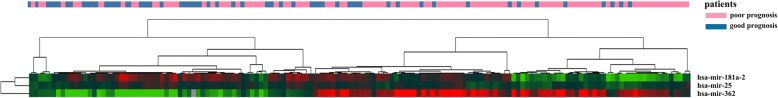
Heat map of selected 3 miRNAs in AML patients. The columns represent patients and rows represented miRNA signature. The green represent relatively lower expression while red represent relatively higher expression

After selection of the three miRNAs, unsupervised hierarchical clustering analysis was conducted based on expression patterns for the AML patients. We found that samples could be separated into two groups (i.e. good prognosis vs. poor prognosis) according to expression levels of the 3 miRNAs (Fig. 3).

**Fig. 4:**
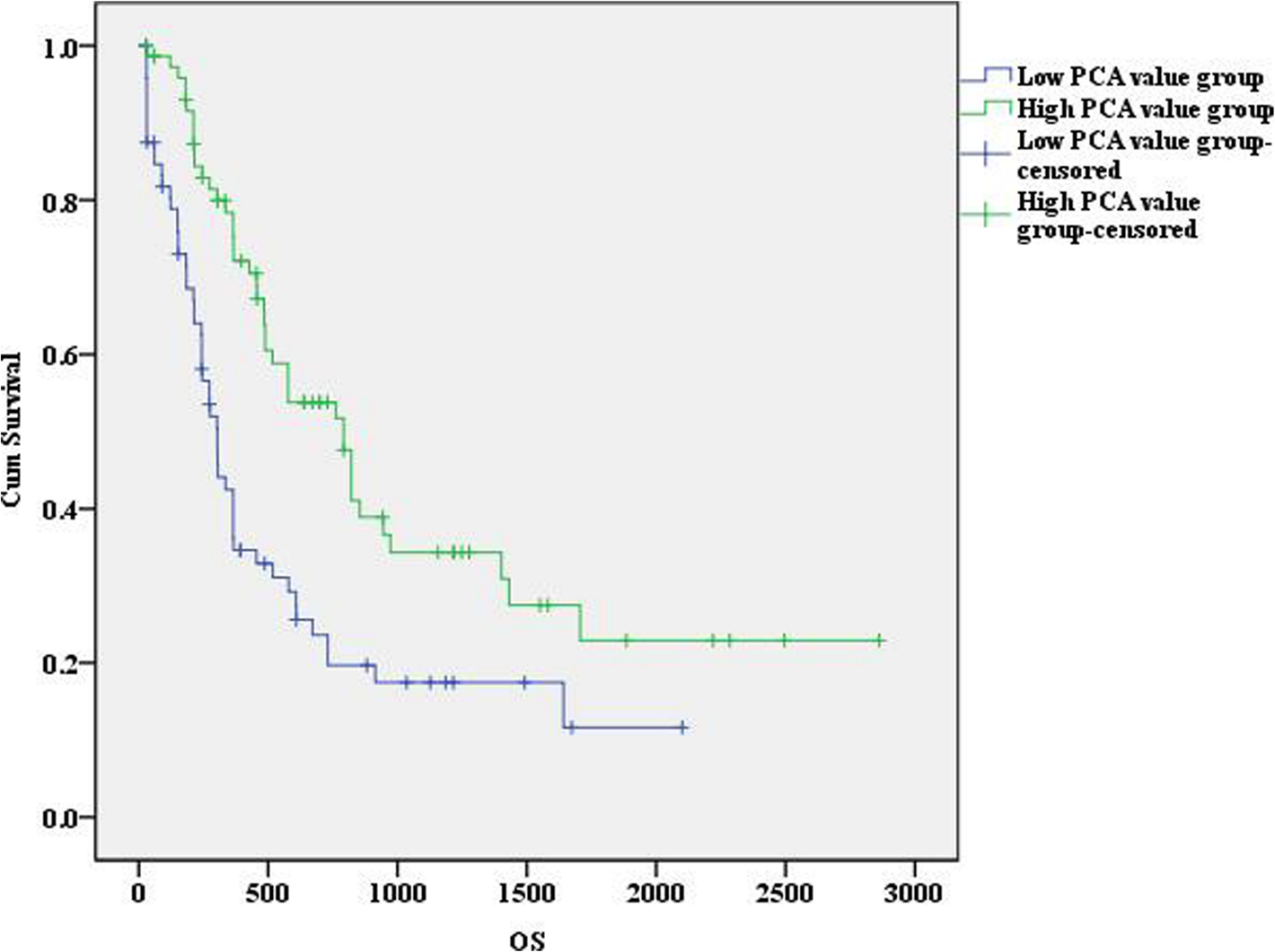
The overall survival of AML patients in different PCA groups, shown by Kaplan-Meier curves. Groups were identified according to patients’ PCA values of the 3 miRNA signatures. The *P*-value of log-rank test for differences between the high-PCA and low-PCA groups was less than 0.001

**Fig. 5:**
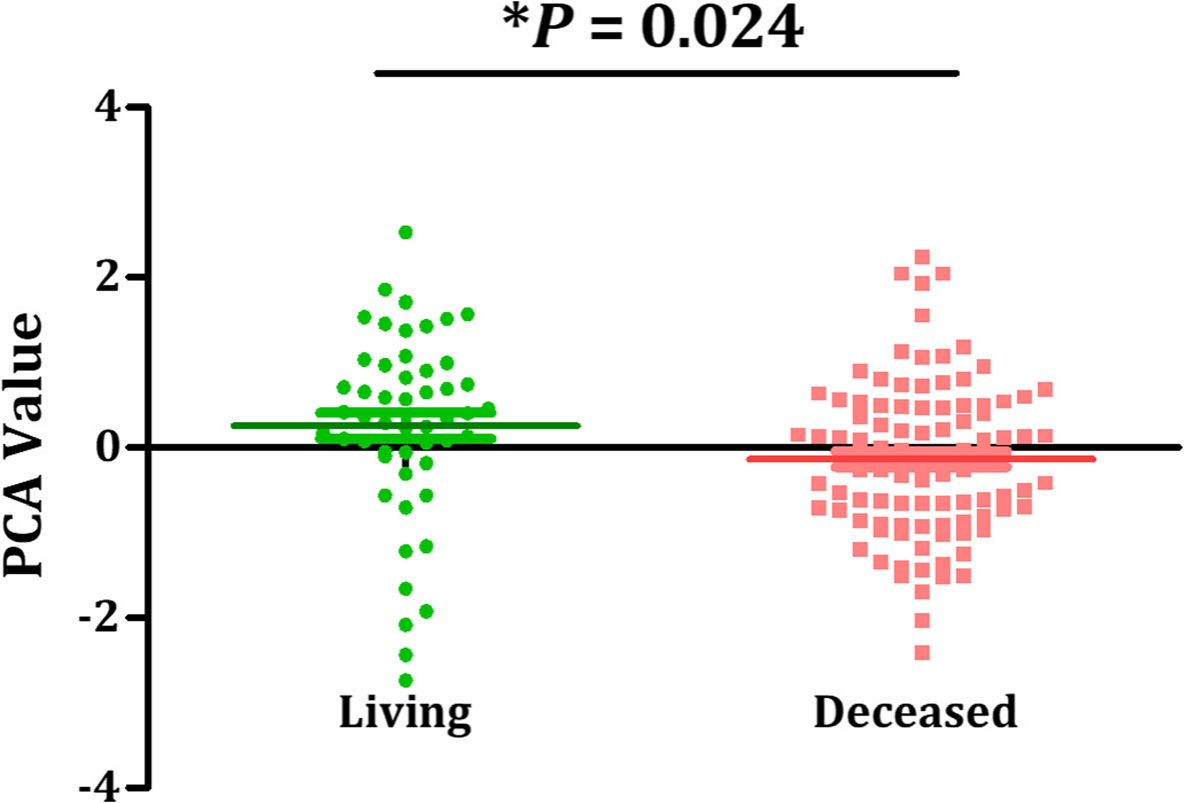
Difference of PCA values between survival AML patients and dead patients

Subsequently, we performed PCA analyses to comprehensively represent the expression levels of the three miRNAs of each patient with a PCA value. We subsequently separated all patients into one of two groups with a cut off set as the median of all PCA values. According to log-rank test, we found that the AML outcome for patients with higher PCA values was significantly different from those with lower PCA values (*P* < 0.001). OS of the two groups is graphical displayed by a Kaplan-Meier curve (Fig. 4). AML patients with lower PCA values, which were derived from comprehensive expression levels of the three selected miRNAs, exhibit significantly poorer prognosis than patients with lower PCA values. Furthermore, difference of PCA values between survival AML patients and dead patients was shown in Fig. 5 (*P* = 0.024), supporting a reliable role of PCA value in predicting AML prognosis.

### Correlated genes and bioinformatic analyses

**Fig. 6:**
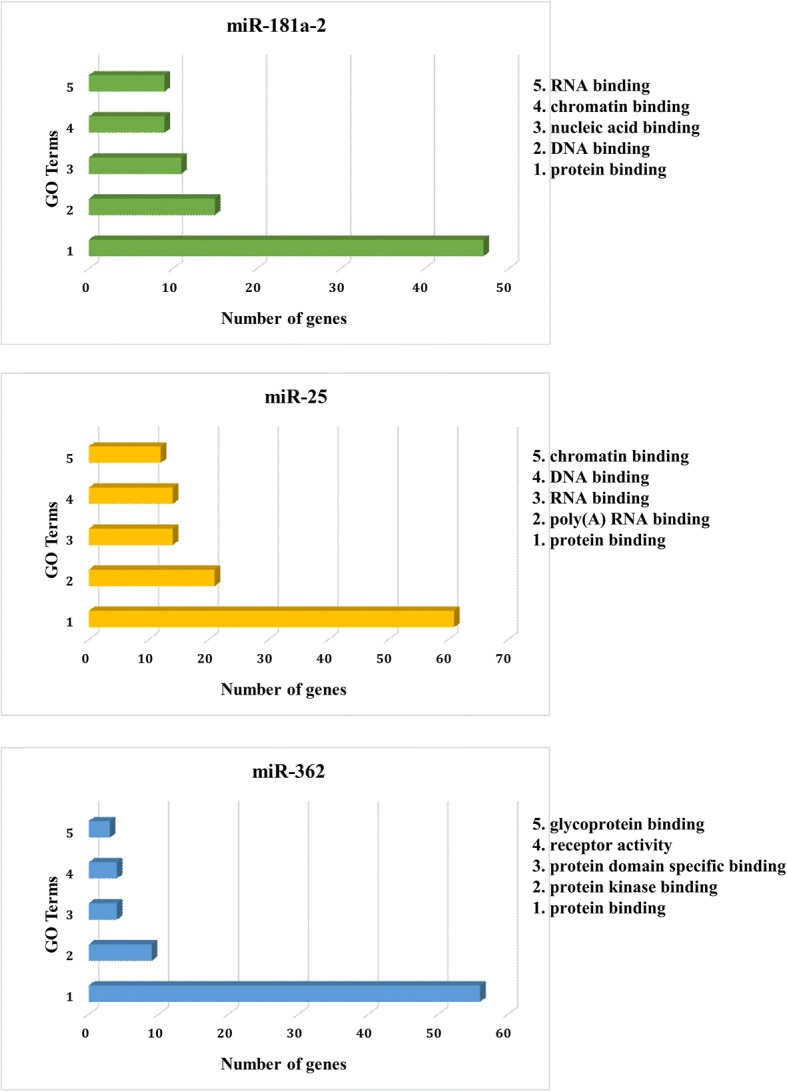
GO terms (molecular Function) of the top 100 correlated coding genes of selected 3 miRNAs

Pearson correlation analysis was performed using TCGA coding gene expression data as well as expression levels of the three miRNAs to identify the most correlated coding genes and understand the potential biological functions of these miRNAs. Top 5 GO terms (molecular function) for the top 100 correlated coding genes for each miRNA are shown in Fig. 6. According to these bioinformatic analyses, we inferred that these miRNAs may be involved in AML prognosis via the regulation of various biological process, including RNA binding (miR-181a-2 and miR-25), chromatin binding (miR-181a-2 and miR-25), protein binding (miR-181a-2, miR-25 and miR-362) etc.. Additional file 2: Table S2 lists the top 10 coding genes correlated with each miRNA. In order to test the reliability of relationship between miRNAs and correlated coding genes (i.e. top coding genes with expression level correlated with each miRNAs), we conducted bioinformatics analyses using Targetscan (http://www.targetscan.org/vert_72/). Finally, we confirmed that many of the TOP 10 genes have binding site for the correlated miRNAs. For instance, a total of 5 genes (i.e. FCF1, PAIP1, MTPAP, ZNF124 and RNASEH2B) listed in Additional file 2: Table S2 have binding site of miR-25.

Furthermore, enriched Kyoto Encyclopedia of Genes and Genomes (KEGG) pathways of correlated coding genes are shown in Table 4. It should be noted that correlated genes of miR-181a-2 and miR-25 enriched in the same pathways (i.e. transcriptional misregulation in cancer, glutamatergic synapse and microRNAs in cancer).

**Table 4:** Relative KEGG pathway of the top 100 correlated coding genes of selected three miRNAs

miRNA	KEGG Pathway	*P* value
miR-181a-2	Transcriptional misregulation in cancer	3.32E-03
	Glutamatergic synapse	6.78E-02
	MicroRNAs in cancer	8.98E-02
miR-25	Transcriptional misregulation in cancer	3.32E-03
	Glutamatergic synapse	6.78E-02
	MicroRNAs in cancer	8.98E-02
miR-362	Mineral absorption	2.47E-03
	Tuberculosis	2.02E-02
	Legionellosis	4.05E-02
	Glycolysis / Gluconeogenesis	5.96E-02
	Phagosome	6.16E-02
	Pertussis	7.27E-02
	Chemokine signaling pathway	9.73E-02

## Discussion

In past decades, numerous studies have investigated biomarkers for the prognosis of cancers. Among various kinds of biomarkers, miRNAs have attracted increasing attention due to their stability in tumour tissue and the ease with which they can be detected in peripheral blood [11–13]. The pathogenesis and prognosis of many cancers has implicated the deregulation of miRNAs, which play vital roles in regulating the expression of coding genes involved in a wide range of biological processes [14, 15]. Therefore, several studies have investigated the clinical value of miRNAs for the prediction of cancer outcomes [9, 16, 17]. Koolivand et al. observed that miR-155 might function as an oncomir in AML and can be a prognosis biomarker for AML [18], while Xu H et al. have reported role for miR-135a in predicting poor prognosis in AML [19]. We inferred from these studies that miRNAs can be valuable biomarkers for the prediction of AML prognosis. However, most current studies have focused on single miRNAs, while systematic analyses of association between whole miRNA expression profiles and AML outcomes remain relatively rare.

In the present study, we obtained data for miRNAs and coding gene expression microarrays in AML patients from the TCGA database. Clinical demographics, including age, gender, risk category, FAB morphology category, as well as OS time and event status, were also downloaded. Analyses showed that patient age and risk category were significantly related to survival status. Previous studies have demonstrated that elderly patients have poorer AML outcomes, partly due to their poorer tolerance to chemotherapy treatment and their reluctance to accept intensive regimens [20, 21]. Our results also showed that the percentage of living patients was remarkably lower in the older group than in the younger group. In addition, we found that the frequency distribution of living patients was significantly different among groups of AML risk categories, which were mainly distinguished by genetic abnormalities [22]. Our results are in accordance with the current consensus that risk stratification of AML is an important prognostic factor [23, 24].

Subsequently, three miRNAs were identified to be significantly associated with AML survival. We subsequently performed PCA analyses to extract data for the expression levels of the three miRNAs, representing them as one PCA value for one each patient. Subsequently, analysis suggested that PCA values can separate AML into two groups with significantly different prognoses. Compared with most studies that identified single miRNAs associated with AML outcome [5, 25], our results are more comprehensive and illustrate the clinical value of a panel of three miRNAs that predict AML survival. In further studies, a scoring system will be constructed from expression level of these three miRNAs to predict prognosis of AML in a quantitative manner.

Recently, Chuang MK identified a 3-microRNA scoring system for prognostication of AML [6], and their list of miRNA signatures (i.e., miR-9, miR-155, miR-203) was different from ours, which were derived from the TCGA study [10]. We speculated this difference was partly due to different populations (i.e., Chuang’s study was conducted in a Taiwanese population). Furthermore, differential analysis methods could also be a reason for the discrepancy.

In addition to this, Gao HY et al. have reported another work identifying some miRNAs associated with AML prognosis, using TCGA data. We have compared our present result with Gao HY’s report [26] and found that only miR-362was in common. However, patients of all AML subtypes were included in Gao’s study, while only non-M3 patients were included in ours because AML-M3 patients have remarkable different biologic features and good outcome from other AML subtypes. Besides, in Gao’ study, they drawn KM curve for each miRNA and miRNAs exhibiting significant differences were selected as candidate factors, while we performed multivariate Cox’s analysis to identify potential miRNAs associated with AML survival. Therefore, we inferred that different study populations as well as different analyse method may be the reason for differences between Gao’s study and ours. On the other hand, we have noticed that miR-362 was identified in both our and Gao’s study, with the same variation trend (i.e. higher expression level predicted poorer prognosis). After a literature reviewing, we found Jin J et al. have also reported high miR-362 expression is associated with poorer OS in AML [27]. There observations provided strong evidence for the outcome predicting value of miR-362 in AML.

With respect to the other miRNAs identified to be valuable predictors of AML outcome, their biological role in cancer prognosis was also investigated through a literature review. As for miR-181a-2, a multiple centre study have demonstrated that higher miR-181a expression was significantly associated with better outcome of AML patients [28]. In xenograft mouse models, ectopic miR-181a expression have also shown to inhibits tumor growth [29]. This miRNA also plays determinant role in the sensitization of leukemic resistant cells to DNR and NK cell [30]. Besides, miR-181a has shown to sensitize resistant leukemia HL-60/Ara-C cells to Ara-C by inducing apoptosis [31]. While for miR-25, one study conducted by Wang Y et al. has reported that it was significantly associated with overall survival of 53 AML patients and its expression was associated with a good outcome [32]. These studies provide further evidence for biological role of miR-181a-2 and miR-25 in AML progression.

Subsequently, bioinformatic analyses were performed to further reveal potential molecular functions and biological pathways for these three miRNAs. Firstly, we tested the reliability of relationship between miRNAs and correlated coding genes by conducting bioinformatics analyses using Targetscan, and further biological experiment will be conducted to confirm the association between other miRNAs and coding genes correlated with them. Then GO analysis revealed molecular activities of the genes most correlated with each miRNA. We found the correlated coding genes for the three miRNAs were relatively concentrated in RNA binding, chromatin binding and protein binding. Therefore, we can infer the biological function of the selected three miRNAs more rationally. In addition, pathway analyses also revealed that correlated coding gene expression for the identified miRNAs participated in transcriptional misregulation in cancer. Some of the potentially related pathways identified were interesting, such as mineral absorption. Previous studies have already shown that abnormalities in mineral homeostasis are involved with acute leukemia [33], but a detailed mechanism for this has not been demonstrated. It should be also noted that correlated genes of miR-181a-2 and miR-25 enriched in the same pathways, indicating a similar mechanism of the two miRNAs in pathogenesis of AML. Recently, Chang H et al. identified robust survival sub-pathways in AML using sample-matched miRNA and gene expression data sets from the TCGA database. Our results are different from their study partly because of the divergent study design, and the pathways we revealed may be a valuable clue for AML exploration. The results of the bioinformatics analyses provided us potential clues for further investigation of the biological function of the three identified miRNAs.

Although our present study have identified potential target genes and related pathways of these 3 miRNAs by bioinformatic approaches, in-depth mechanical approaches are still needed to verify our bioinformatic result. This is a limitation of our study. However, some of the top correlated coding genes have already been reported to be mutated in AML (e.g. CCND2, STMN1 and TFEB), which provided indirect evidence for our prediction that the selected 3 miRNAs may participate in AML by targeting these “top genes” [34–36]. In addition, the case numbers of TCGA data downloaded were too small to be split into training and validation cohorts. This is a limitation of our present study. In the future, we will collect more AML samples to create a validation cohort for our results.

## Conclusions

In conclusion, the present study identified three miRNAs through comprehensive analysis of TCGA AML data that are significantly related with AML survival. PCA values of the identified miRNAs play important roles assessing the predictive values of these miRNAs for AML prognosis. Further studies focused on a scoring system to predict AML survival should be conducted.

## Additional files

Additional file 1: **Table S1.** Title of data: List of top 50 miRNAs in the multivariate analysis after adjustment by FDR. Description of data: This Table listed TOP 50 miRNAs after multivariate analysis and the top 3 miRNAs were significantly associated with AML prognosis. (DOCX 18 kb)

Additional file 2: **Table S2.** Title of data: Top ten coding genes correlated with each selected miRNAs. Description of data: This table listed top 10 coding genes associated with the 3 selected miRNAs. This correlation was analyzed by Pearson correlation and the expression level of top 10 coding genes was associated with 3 miRNAs with the highest Pearson correlation coefficient. (DOCX 16 kb)

## Abbreviations

AML: Acute myeloid leukemia
DAVID: Database for annotation, visualization and integrated discovery
FDR: False discovery rate
GO: Gene ontology
GO: Gene ontology
KEGG: Kyoto Encyclopedia of Genes and Genomes
miRNAs: microRNAs
NCCN: National Comprehensive Cancer Network
OS: Overall survival
PCA: Principal component analysis
TCGA: The Cancer Genome Atlas
WBC: White blood cell
