# Supplementary material for: Selection of three miRNA signatures with prognostic value in non-M3 acute myeloid leukemia
Source: BMC Cancer. 2019 Jan 30;19:109. doi: 10.1186/s12885-019-5315-z (PMC6483142; doi:10.1186/s12885-019-5315-z)
Supplement: Supplementary file 1 — Table S1. Title of data: List of top 50 miRNAs in the multivariate analysis after adjustment by FDR. Description of data: This Table listed TOP 50 miRNAs after multivariate analysis and the top 3 miRNAs were significantly associated with AML prognosis. (DOCX 18 kb) [file 12885_2019_5315_MOESM1_ESM.docx]

Table S1. List of top 50 miRNAs in the multivariate analysis after adjustment by FDR

| miRNAs | P value | FDR | Hazard Ratio |
| --- | --- | --- | --- |
| mir.181a.2 | 2.52E-04 | 4.69E-02 | 0.45 |
| mir.25 | 3.98E-04 | 4.69E-02 | 0.46 |
| mir.362 | 3.84E-04 | 4.69E-02 | 2.13 |
| mir.106b | 2.21E-03 | 1.22E-01 | 0.53 |
| mir.20b | 3.09E-03 | 1.22E-01 | 2.11 |
| mir.502 | 2.64E-03 | 1.22E-01 | 1.89 |
| mir.532 | 2.81E-03 | 1.22E-01 | 1.90 |
| mir.181c | 3.01E-03 | 1.22E-01 | 0.53 |
| mir.636 | 2.94E-03 | 1.22E-01 | 1.87 |
| mir.501 | 3.99E-03 | 1.33E-01 | 1.83 |
| mir.451 | 4.13E-03 | 1.33E-01 | 1.90 |
| mir.486 | 6.11E-03 | 1.44E-01 | 1.96 |
| mir.30d | 5.02E-03 | 1.44E-01 | 0.55 |
| mir.188 | 6.05E-03 | 1.44E-01 | 1.80 |
| mir.363 | 5.35E-03 | 1.44E-01 | 1.98 |
| mir.500 | 8.76E-03 | 1.84E-01 | 1.74 |
| mir.550.1 | 8.84E-03 | 1.84E-01 | 0.57 |
| mir.660 | 1.05E-02 | 2.06E-01 | 1.71 |
| mir.100 | 1.11E-02 | 2.06E-01 | 0.59 |
| mir.1304 | 1.28E-02 | 2.27E-01 | 1.69 |
| mir.195 | 1.66E-02 | 2.81E-01 | 0.60 |
| mir.181d | 2.35E-02 | 3.46E-01 | 0.62 |
| mir.135b | 2.33E-02 | 3.46E-01 | 0.60 |
| mir.199a.1 | 2.33E-02 | 3.46E-01 | 0.61 |
| mir.107 | 2.82E-02 | 3.89E-01 | 1.62 |
| mir.181a.1 | 2.96E-02 | 3.89E-01 | 0.64 |
| mir.181b.1 | 2.96E-02 | 3.89E-01 | 0.63 |
| mir.331 | 3.19E-02 | 4.03E-01 | 0.63 |
| mir.378 | 3.34E-02 | 4.07E-01 | 1.60 |
| mir.93 | 3.89E-02 | 4.60E-01 | 0.64 |
| mir.20a | 4.32E-02 | 4.93E-01 | 1.53 |
| mir.149 | 4.57E-02 | 5.00E-01 | 1.52 |
| mir.101.1 | 4.66E-02 | 5.00E-01 | 0.66 |
| mir.1266 | 4.92E-02 | 5.13E-01 | 0.65 |
| mir.579 | 5.28E-02 | 5.34E-01 | 1.50 |
| mir.183 | 5.63E-02 | 5.54E-01 | 1.51 |
| mir.618 | 6.92E-02 | 6.13E-01 | 0.68 |
| mir.412 | 6.65E-02 | 6.13E-01 | 1.48 |
| mir.1468 | 6.78E-02 | 6.13E-01 | 0.68 |
| mir.607 | 6.81E-02 | 6.13E-01 | 1.50 |
| mir.200c | 7.20E-02 | 6.22E-01 | 1.47 |
| mir.133b | 8.10E-02 | 6.51E-01 | 1.44 |
| mir.224 | 8.01E-02 | 6.51E-01 | 0.69 |
| mir.766 | 7.79E-02 | 6.51E-01 | 0.68 |
| mir.133a.2 | 8.29E-02 | 6.52E-01 | 1.46 |
| mir.1826 | 8.86E-02 | 6.82E-01 | 0.70 |
| mir.1248 | 1.27E-01 | 6.85E-01 | 0.73 |
| mir.142 | 1.37E-01 | 6.85E-01 | 0.73 |
| mir.581 | 1.20E-01 | 6.85E-01 | 0.72 |
| mir.320d.2 | 1.08E-01 | 6.85E-01 | 0.71 |
