# Supplementary material for: Selection of three miRNA signatures with prognostic value in non-M3 acute myeloid leukemia
Source: BMC Cancer. 2019 Jan 30;19:109. doi: 10.1186/s12885-019-5315-z (PMC6483142; doi:10.1186/s12885-019-5315-z)
Supplement: Supplementary file 2 — Table S2. Title of data: Top ten coding genes correlated with each selected miRNAs. Description of data: This table listed top 10 coding genes associated with the 3 selected miRNAs. This correlation was analyzed by Pearson correlation and the expression level of top 10 coding genes was associated with 3 miRNAs with the highest Pearson correlation coefficient. (DOCX 16 kb) [file 12885_2019_5315_MOESM2_ESM.docx]

Table S2. Top ten coding genes correlated with each selected miRNAs.

| miRNAs | Top 10 correlated miRNAs | |
| --- | --- | --- |
| miR-181a-2 | FLJ22536 | ZNF608 |
|  | USP13 | GNG7 |
|  | MEX3C | CCND2 |
|  | C12orf23 | C11orf95 |
|  | ZNF124 | ARPP21 |
| miR-25 | STMN1 | PAIP1 |
|  | KHDRBS1 | NUFIP1 |
|  | TSGA14 | MTPAP |
|  | FCF1 | ZNF124 |
|  | PM20D2 | RNASEH2B |
| miR-362 | CLCN5 | CHMP4B |
|  | C11orf75 | TFEB |
|  | KIF1C | HNMT |
|  | METTL7B | PEA15 |
|  | S100A6 | PSTPIP1 |
